# Neurophysiological evidence for emotion regulation abnormalities in individuals at clinical high-risk for psychosis

**DOI:** 10.1007/s00406-025-02006-y

**Published:** 2025-04-26

**Authors:** Gregory P. Strauss, Lisa A. Bartolomeo, Gifty Ayawvi

**Affiliations:** Department of Psychology, University of Georgia, 125 Baldwin St, Athens, GA 30602 USA

**Keywords:** Prodrome, Attenuated psychosis syndrome, Ultra-high-risk, Schizophrenia, EEG, Late positive potential

## Abstract

Past studies indicate that individuals at clinical high-risk for psychosis (CHR) display emotion regulation abnormalities that predict increased symptom severity and poor functional outcome. However, it is unclear which neurophysiological processes contribute to impairments in implementing various strategies to down-regulate negative emotion. The current study used electroencephalography (EEG) to determine whether individuals at CHR have difficulty implementing reappraisal and distraction. Participants included individuals at CHR (*n* = 25) and healthy controls (CN: *n* = 36) who completed an EEG task while unpleasant or neutral stimuli were presented and they were required to either passively view or down-regulate negative emotion using reappraisal or distraction. The late positive potential (LPP) event related potential component was calculated from the EEG data and used as an objective neurophysiological indicator of emotion regulation effectiveness. CN effectively decreased the amplitude of the LPP for both reappraisal and distraction compared with unpleasant passive viewing; however, CHR did not differ in LPP amplitude for unpleasant passive viewing, reappraisal, and distraction, suggesting an implementation abnormality. Difficulty implementing distraction was associated with greater severity of attenuated positive symptoms. Collectively, these findings suggest that CHR display neurophysiological patterns of emotion regulation impairment that are similar to those that have been identified among individuals with schizophrenia in past studies. Interventions have been developed to target these mechanisms. It may be beneficial to apply these interventions to psychosis-spectrum populations where they would have relevance for both treatment of established symptoms and prevention of illness among those at CHR.

## Introduction

Numerous studies support the role of elevated negative emotion or stress reactivity in the onset and maintenance of psychotic disorders (PDs) [8, 40, 41]. Emerging evidence also indicates that impairments in implementing emotion regulation strategies to control the negative emotion/stress response have an additive effect above and beyond heightened stress reactivity in contributing to psychosis risk. For example, past studies using trait emotion regulation questionnaires suggest that similar to those with full PDs [6, 14, 17, 23, 36,], individuals at clinical high-risk for psychosis (CHR) (i.e., those meeting criteria for a psychosis risk syndrome) [6, 18, 39] report being less likely to utilize adpative emotion regulation strategies (e.g., reappraisal) and more likely to attempt maladaptive strategies (e.g., suppression) than healthy controls (CN). Furthermore, greater self-reported use of maladaptive strategies and reduced use of adaptive strategies predicts numerous poor clinical outcomes in CHR (e.g., higher positive, negative, and general symptoms; poor functioning) [6, 18, 39], similar to what is observed in SZ [2, 6, 14, 17, 21, 36]. These findings suggest that emotion regulation abnormalities may be a critical treatment target across phases of psychotic illness.

However, trait self-reports alone provide limited insight into which components of emotion regulation abnormalities should be targeted. To address this issue, several laboratory-based studies have examined emotion regulation using methods and conceptual frameworks from the fields of cognitive and affective neuroscience. The majority of these studies have used electroencephalography (EEG) to determine whether individuals with PDs can effectively decrease the neurophysiological response to unpleasant stimuli using an instructed strategy (e.g., reappraisal) compared to passively viewing unpleasant stimuli. A key outcome measure from these studies is the late positive potential (LPP), a midline centroparietal event related potential (ERP) component that emerges at approximately 300ms after stimulus onset and persists throughout the duration of stimulus exposure [13]. During passive viewing, healthy individuals and those with PDs have higher amplitude LPPs than CN for unpleasant than neutral stimuli (i.e., intact emotional reactivity). However, individuals with PDs are less effective at implementing a variety of emotion regulation strategies (e.g., reappraisal, directed attention, distraction) to reduce LPP amplitude while viewing unpleasant stimuli compared to CN [4, 14, 31–33]. fMRI studies using similar paradigms suggest that these neurophysiological emotion regulation abnormalities may result from reduced activation of key regions within the prefrontal cortex when implementing reappraisal (e.g., ventrolateral prefrontal cortex, anterior cingulate cortex, anterior cingulate cortex, orbitofrontal cortex) [20, 26, 37, 38, 42]. It is unclear whether these same processes contribute to emotion regulation abnormalities in CHR and its associated attenuated symptoms and functional impairment.

In the current study, we administered an EEG/ERP paradigm previously applied to study reappraisal and distraction in PDs [4] to a sample of CHR participants and matched CN. Based on our past findings in PDs, the following hypotheses were made: (1) CHR will display subjective emotion regulation impairments, as indicated by significantly higher self-reported levels of negative emotion during the reappraisal and distraction conditions than CN; (2) CHR will display a neurophysiological emotion regulation abnormality compared to CN, as indicated by greater difficulty decreasing the LPP while attempting to implement reappraisal and distraction emotion regulation strategies; (3) Emotion regulation abnormalities measured via the LPP will be associated with greater severity of positive symptoms, negative symptoms, and poorer functioning.

## Method

### Participants

Participants included individuals at clinical high-risk for psychosis (CHR: *n* = 25) and psychiatrically healthy controls (CN: *n* = 36). CHR participants were recruited from the Georgia Psychiatric Risk Evaluation Program (G-PREP) that received referrals from local clinicians to perform diagnostic assessment and monitoring evaluations for youth displaying psychotic experiences. Additional recruitment methods included online and print advertisements, in-person presentations to community mental health centers, and calls or meetings with members of the local school system. CHR met criteria for a prodromal syndrome on the Structured Interview for Prodromal Syndromes (SIPS) [25]. None of the CHR participants met lifetime criteria for a DSM-5 psychotic disorder on the SCID-5 [9].

CN were recruited from the local community using posted flyers and electronic advertisements. CN had no current major psychiatric disorder diagnoses and no SZ-spectrum personality disorders as established by the SCID-5 [9] and SCID-5-PD [10], no family history of psychosis, and were not taking psychotropic medications. All participants were free from lifetime neurological disease.

Groups did not significantly differ on age, race, sex, personal education, or parental education (see Table 1).

**Table 1:** Demographic characteristics

	CHR (*n* = 25)	CN (*n* = 36)	Test Statistic	*p*-value
Age	22.36 (2.69)	21.56 (3.08)	*F =* 1.11	0.30
Parental Education	15.27 (2.35)	16.02 (2.81)	*F =* 1.19	0.28
Participant Education	14.40 (1.71)	14.19 (1.60)	*F =* 0.24	0.63
% Female	80.0	72.2	χ^2^ = 0.28	0.56
Race (%)			χ^2^ = 3.20	0.67
White	68.0	72.2		
Black	4.0	2.8		
Asian-American	16.0	11.1		
Hispanic/Latino	8.0	8.3		
Biracial	0	5.6		
Other	4.0	0		

Note. CHR = clinical high-risk for psychosis; CN = healthy control

### Procedure

All participants provided informed consent in accordance with the UGA Institutional Review Board and the study was performed in accordance with the ethical standards laid down in the 1964 Declaration of Helsinki and its later amendments. Research staff trained to reliability standards conducted structured clinical interviews to assess diagnostic status, including the SCID-5 and the SIPS. Functioning was assessed using the Global Functioning Scale: Social [1] and Role [27]. Following the completion of clinical interviews, participants came to the lab for an in-person session to complete the EEG emotion regulation task.

#### EEG emotion regulation task

The EEG emotion regulation paradigm was modeled after Thiruchselvam et al. [35]. The task consisted of 112 trials completed over 2 blocks, each containing 56 trials where each trial consisted of a unique image presented only once during the task. Conditions included: Watch (passively viewing an unpleasant image), View (passively viewing a neutral image), Reappraise (reinterpret the meaning of the image so it is less negative), and Distract (think of a complex geometric object or neutral scene to feel less negative). On View and Watch trials, participants were instructed to look at the image and allow their thoughts and emotions to unfold naturally. Cues with condition instructions were also presented with distinct background colors: black for View, grey for Watch, blue for Distract, and green for Reappraise.

At the beginning of each trial, participants attended to a central white fixation cross against a black screen for 1 s. Next, an instruction cue was presented for 2 s, after which an IAPS image appeared for 5 s. Following each image, unlimited time was given to report subjective level of negative emotion on a unipolar 1–5 scale (i.e., 1 = *Not at all* to 5 = *Extremely*) using the Self-Assessment Manikin via gamepad response.

Each block included 14 View and 14 Watch trials, and either 28 Distract or 28 Reappraise trials. Reappraisal and distract blocks were administered separately and in counterbalanced order to reduce task demands (e.g., switching between strategies between trials) and increase chances that participants would apply the strategy as instructed by the cue on each trial. Trial order was randomized within blocks. Unpleasant and neutral images significantly differed on valence and arousal using normative IAPS rating data. Unpleasant stimuli presented in reappraise, distract, and unpleasant passive viewing conditions did not significantly differ on valence or arousal on IAPS norms.

#### EEG recording and data reduction procedures

EEG was recorded from a subset of Ag/AgCl electrodes mounted in a 64-channel BrainVision ActiCap. Signals were recorded online using a right mastoid reference and re-referenced offline to the average of the left and right mastoids. All electrode impedances were maintained below 15KΩ. The EEG signal was amplified via a BrainVision actiCHamp amplifier with gain of 5000, bandpass filter of 0.05–100 Hz, and 60-Hz notch filter. Amplified signals were digitized at 500 Hz and averaged offline.

Matlab and the EEGLAB/ERPLAB toolboxes were used for signal processing [24]. Large muscle artifacts and extreme offsets were identified by visual inspection and removed. Independent component analysis (ICA) was conducted on the continuous data to identify and correct eyeblink activity. The EEG was high-pass filtered with a cut-off of 0.1 Hz.

#### ERP measurement procedures

ERPs were constructed by taking the grand average of all trials within the four conditions (Unpleasant Passive, Neutral, Reappraise, Distract). The LPP was measured across electrode sites Cz, CP1, CP2, and Pz, as the average amplitude between 300-5000ms post-stimulus onset. Measurement procedures were consistent with prior work in this area and this particular task [4, 13, 31, 33].

#### Data analysis

Within group paired samples *t*-tests were used to determine whether emotional reactivity was intact for each group (i.e., Unpleasant Passive > Neutral). Mixed models ANOVAs were used to evaluate primary hypotheses regarding interactions between Group and task condition. Significant effects were followed up by post hoc contrasts. Sensitivity power analysis indicated that given the sample size (*n* = 61) and power, the primary analysis (LPP group x condition interaction) was powered to detect a medium effect (effect size f = 0.13; critical f = 2.65, non-centrality parameter = 17.55).

Bivariate Spearman correlations were used to evaluate hypothesized associations between experimental and clinical variables. Difference scores (Unpleasant Passive - Distract or Reappraise) were calculated for use in correlations to isolate emotion regulation effects specifically. The Benjamini and Hochberg method was used to correct for multiple comparisons.

## Results

### Self-Report results

**Fig. 1:**
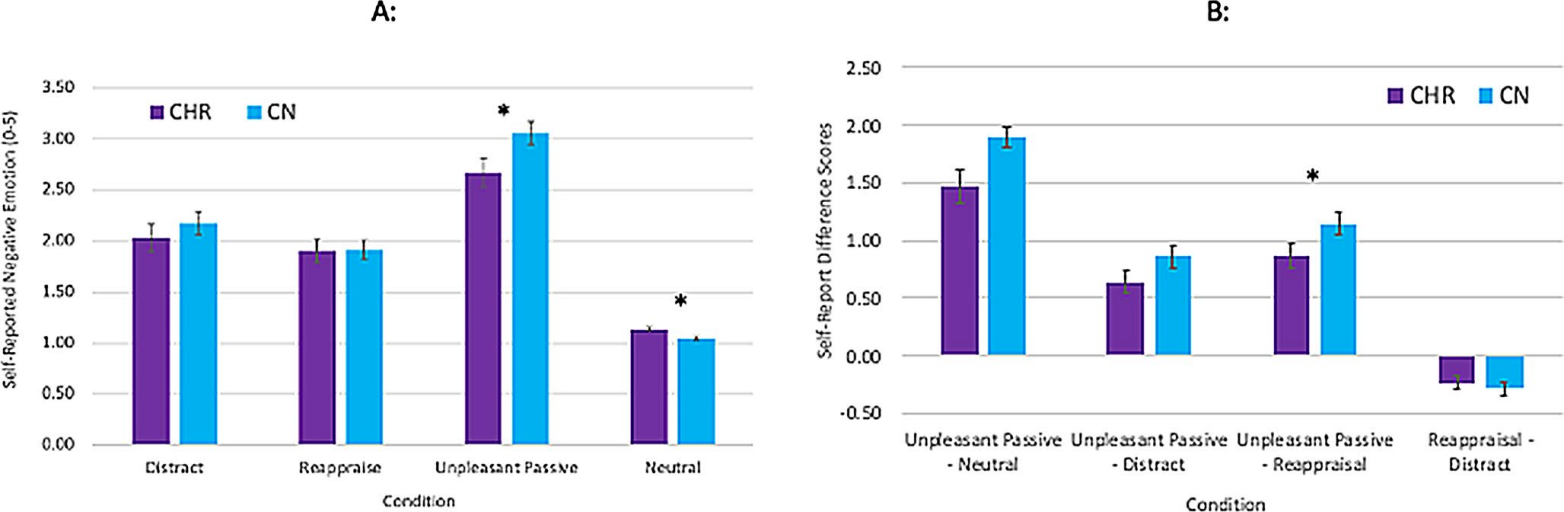
Self-reported emotional experience in CHR and CN groups. Note. Panel A = Raw scores; Panel B = difference scores; CHR = clinical high-risk for psychosis; CN = healthy control

Within group paired samples *t*-tests indicated that both groups showed higher self-reported negative emotion for unpleasant passive viewing (*M*_CN_ = 3.06, *SD*_CN_ = 0.59; *M*_CHR_ = 2.67, *SD*_CHR_ = 0.79) than neutral stimuli (*M*_CN_ = 1.05, *SD*_CN_ = 0.06, *t* = 21.3, *p* <.001, d = 3.42; *M*_CHR_ = 1.14, *SD*_CHR_ = 0.14, *t* = 9.5, *p* <.001, d = 2.16), consistent with intact emotional reactivity (see Fig. 1).

A 2 Group (CHR, CN) x 4 Condition (Unpleasant Passive, Neutral, Reappraise, Distract) mixed models ANOVA indicated a significant within-subjects effect of condition, F (3, 146.69) = 178.4, *p* <.001 ($\:{\eta\:}_{p}^{2}$ = 0.76), non-significant between-subjects effect of Group, F(1,57) = 1.01, *p* =.30 ($\:{\eta\:}_{p}^{2}$ = 0.02), and significant Group X Condition interaction, F (2.41, 137.14) = 3.64, *p* =.014 ($\:{\eta\:}_{p}^{2}$ = 0.06). Follow-up within-group paired samples t-tests indicated that for both groups, participants reported less negative affect during reappraisal than unpleasant passive viewing (CHR d = 1.76; CN d = 1.97) and distraction than unpleasant passive viewing (CHR d = 1.34; CN d = 1.55) (all p’s < 0.001). One-way ANOVAs calculated using difference scores (Unpleasant Passive – Reappraise or Distract) clarified that groups did not differ in subjective effectiveness of distraction, (F 1, 58) = 2.75, *p* =.10 ($\:{\eta\:}_{p}^{2}$. = 04) [*M*_CN_ = 0.89, *SD*_CN_ = 0.63; *M*_CHR_ = 0.64, *SD*_CHR_ = 0.45] and that reappraisal was significantly less effective in CHR than CN, F (1, 58) = 5.85, *p* =.019 ($\:{\eta\:}_{p}^{2}$. = 06) [*M*_CN_ = 1.15, *SD*_CN_ = 0.64; *M*_CHR_ = 0.77, *SD*_CHR_ = 0.51]. Thus, at the subjective level, emotion regulation was largely intact in CHR but reappraisal was somewhat less effective.

### ERP results

Groups did not significantly differ in the number of usable trials for any of the conditions (M (SD) out of 28 possible trials): Reappraise: CHR = 26.58 (2.65), CN = 27.50 (1.08); Distract: CHR = 27.40 (0.82), CN = 26.72 (3.27); View: CHR = 26.76 (2.82), CN 27.19 (2.11); Watch: CHR = 26.44 (3.27), CN = 27.22 (1.82).

To examine reliability of the LPP, Cronbach’s alpha was calculated for LPP amplitudes across electrode sites Cz, CP1, CP2, and Pz within the 300-5000ms window using the 4 conditions as items and 61 participants as observations (see Thigpen et al., [34]. Cronbach’s alpha was 0.76 for CHR and 0.86 for CN.

Within group paired samples *t*-tests indicated that both groups showed higher LPP amplitude for unpleasant passive viewing (*M*_CN_ = 6.23, *SD*_CN_ = 6.03; *M*_CHR_ = 3.34, *SD*_CHR_ = 4.86) than neutral (*M*_CN_ = 1.17, *SD*_CN_ = 4.62, *t* = 6.67, *p* <.001, d = 1.11; *M*_CHR_ = − 0.66, *SD*_CHR_ = 4.32, *t* = 4.84, *p* <.001, d = 0.97) stimuli, suggesting intact emotional reactivity.

**Fig. 2:**
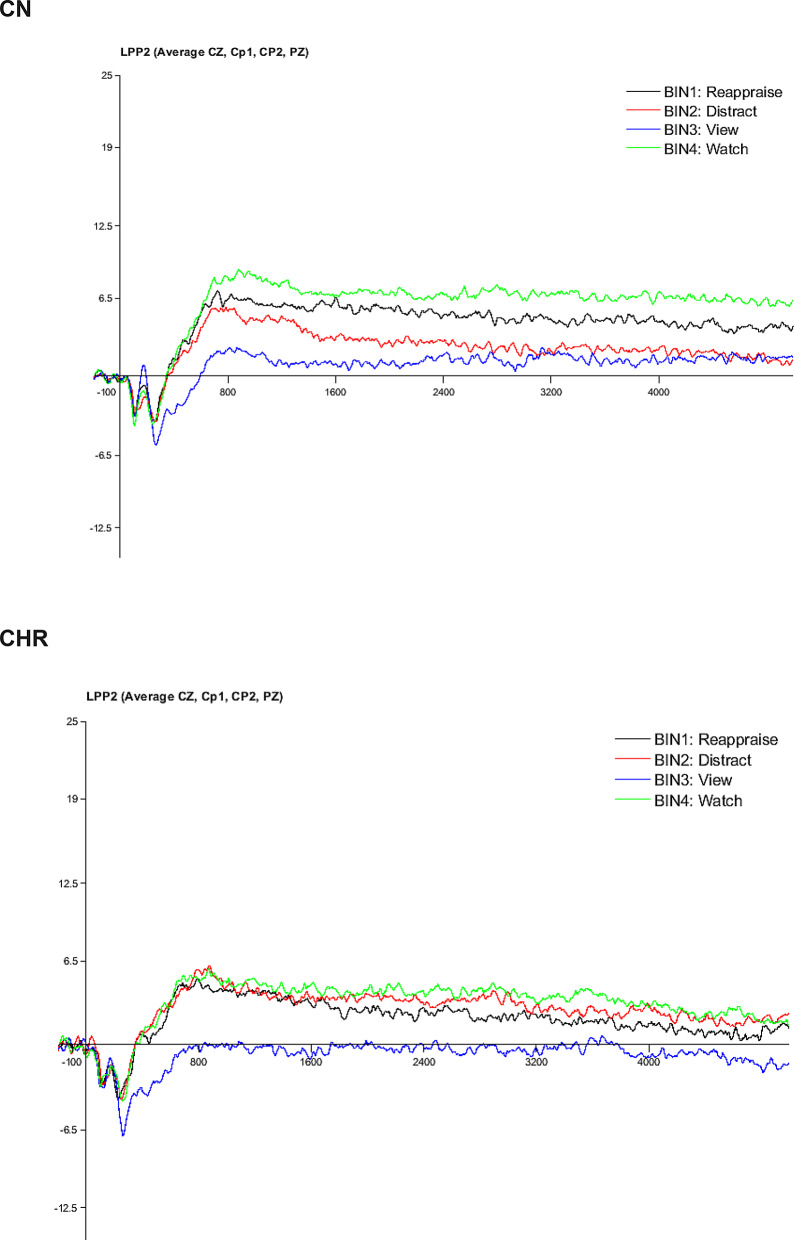
Late positive potential waveforms in CHR and CN groups. Note. CHR = clinical high-risk for psychosis; CN = healthy control

**Fig. 3:**
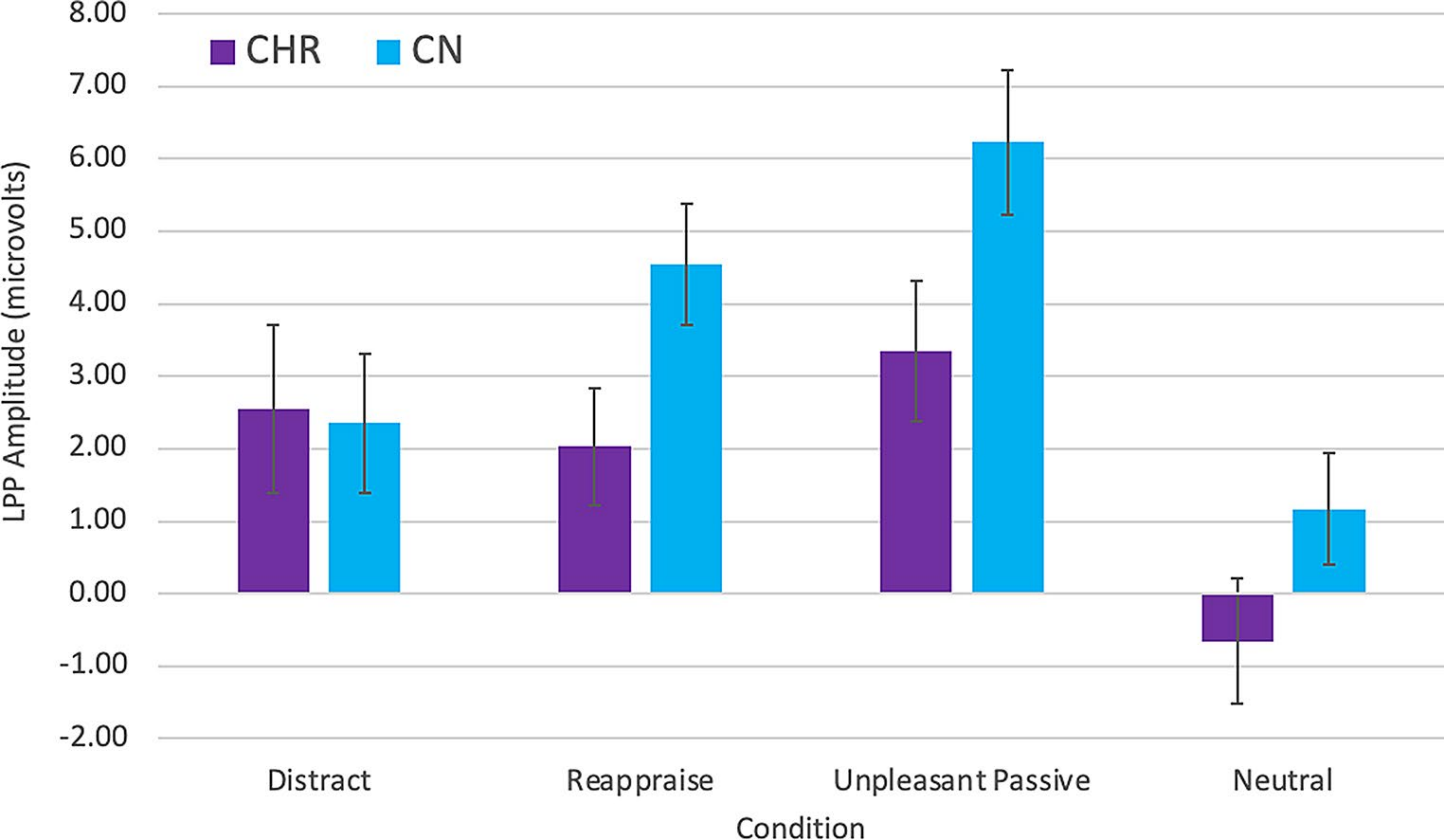
Late positive potential means and standard errors in CHR and CN Groups. Note. CHR = clinical high-risk for psychosis; CN = healthy control; LPP = late positive potential event related potential component

A 2 Group (CHR, CN) x 4 Condition (Unpleasant Passive, Neutral, Reappraise, Distract) mixed models ANOVA indicated a significant main effect of condition, F (3,174) = 20.01, *p* <.001 ($\:{\eta\:}_{p}^{2}$ = 0.26), nonsignificant main effect of group, F (1,58) = 2.45, *p* =.12 ($\:{\eta\:}_{p}^{2}$ = 0.04), and significant group x condition interaction, F (3,174) = 2.70, *p* =.048 ($\:{\eta\:}_{p}^{2}$ = 0.04), for LPP amplitude. Within-group paired samples *t*-tests indicated that CN were effective at implementing both reappraisal and distraction to reduce the neural response to unpleasant stimuli, evidenced by significantly lower LPP amplitude for Reappraise (*M*_CN_ = 4.54, *SD*_CN_ = 5.02; *t* = 2.38, *p* =.02, d = 0.40) and Distract (*M*_CN_ = 2.34, *SD*_CN_ = 5.73; *t* = 4.86, *p* <.001, d = 0.81) conditions compared to Unpleasant Passive. In contrast, CHR were ineffective at implementing both strategies to reduce the neural response to unpleasant stimuli, evidenced by nonsignificant differences between LPP amplitude for both Reappraise (*M*_CHR_ = 2.03, *SD*_CHR_ = 3.95; *t* = 1.17, *p* =.26, d = 0.24) and Distract (*M*_CHR_ = 3.89, *SD*_CHR_ = 5.82; *t* = 0.63, *p* =.54. d = 0.13) conditions compared to Unpleasant Passive (see Figs. 2 and 3). Thus, CHR displayed impaired implementation of both reappraisal and distraction at the neurophysiological level.

### Correlation results

For the Unpleasant Passive – Distract difference score, less modulation of the LPP (i.e., greater emotion regulation impairment) was associated with more severe positive symptoms (ρ = − 0.46, *p* =.02), but not negative symptoms or poor functioning. All correlations with the reappraisal difference score were nonsignificant (see Table 2).

**Table 2:** Correlations between clinical variables and the late positive potential difference scores

	SIPS Positive	SIPS Negative	GFS: S	GFS: *R*
Unpleasant Passive - Reappraisal	− 0.07	− 0.15	0.09	− 0.31
Unpleasant Passive - Distract	− 0.46*	− 0.01	0.07	− 0.24

Note. SIPS = Structured Interview for Prodromal Syndromes; GFS: S = Global functioning scale- social; GFS: R = Global functioning scale- role. * *p* <.05

## Discussion

The primary aim of the current study was to evaluate whether individuals at CHR were less effective at implementing distraction and reappraisal emotion regulation strategies at the neurophysiological levels compared to CN. A secondary aim was to determine whether neurophysiological emotion regulation abnormalities were associated with clinical outcomes in CHR participants.

Consistent with hypotheses, analyses conducted on the ERP data supported the existence of emotion regulation abnormalities in CHR at the neurophysiological level. Specifically, CN were able to decrease LPP amplitude for reappraisal and distraction compared to the unpleasant passive viewing comparison condition. In contrast, CHR did not display differences in LPP amplitude among unpleasant passive viewing, reappraisal, and distraction conditions. Additionally, greater difficulty implementing distraction at the neurophysiological level, but not reappraisal, was associated with greater severity of SIPS positive symptom scores. At the subjective level, emotion regulation was largely intact in CHR; however, CHR were somewhat less effective at implementing reappraisal than CN.

These findings are consistent with prior ERP and fMRI studies indicating that individuals with PDs have impairments in decreasing the neural response to unpleasant stimuli when instructed to use emotion regulation strategies [14, 26, 31, 32, 37, 42]. Evidence regarding subjective emotion regulation effectiveness on tasks has been mixed in PDs, with similar evidence for greater impairments and spared emotion regulation in PDs across studies [4, 19, 31, 32]. Given what is known regarding the neural basis of the LPP [22, 28] and well-documented circuit-level activation patterns that occur during emotion regulation tasks [3] in the general population, these findings may suggest a common neurophysiological mechanism for emotion regulation impairments across phases of psychotic illness in areas such as the ventrolateral prefrontal cortex, insula, and orbitofrontal cortex. Future fMRI or combined fMRI + EEG studies are needed to confirm this possibility.

Findings should be interpreted in light of certain limitations. First, the use of ERPs precludes direct observation of the specific regions of interest and circuits contributing to the neurophysiological abnormalities observed in this study. Second, the study was cross-sectional and implications of these findings for risk of converting to a full psychotic disorder cannot be determined without a prospective longitudinal study. Third, the strategies examined in this study and the overall interpretation of results were grounded in the extended process model of emotion regulation [11]. This model focuses on a small range of possible strategies and explicit modes of emotion regulation. However, other strategies are emphasized by alternate emotion regulation models and there is a growing literature on implicit emotion regulation effectiveness [5, 12]. In future studies, it will be beneficial to study both implicit and explicit emotion regulation for a range of strategies and to incorporate multiple conceptual models.

Despite these limitations, findings have several important implications. First, theories positing a role for heightened stress reactivity in the vulnerability for psychosis [8, 40, 41], may benefit from being updated to include elements of emotion regulation dysfunction. Second, our LPP data suggest that circuits underlying emotion regulation impairment could be novel targets in future interventions. Interventions have been developed that target these neural mechanisms but they have not yet been attempted in the psychosis-spectrum, where they may have relevance for both treatment and prevention [7, 29]. Interventions developed to date have primarily focused on psychological mechanisms (e.g., acceptance) using psychosocial therapies (e.g., mindfulness, acceptance and commitment therapy) [15, 16, 30]. These have generally produced small to medium effect sizes across multiple facets of psychopathology in psychosis (e.g., negative symptoms, social functioning) and may be useful when paired with other interventions targeting neurobiological mechanisms [15].
